# Enhanced Dissolution and Oral Bioavailability of Cyclosporine A: Microspheres Based on αβ-Cyclodextrins Polymers

**DOI:** 10.3390/pharmaceutics10040285

**Published:** 2018-12-18

**Authors:** Malika Lahiani-Skiba, Francois Hallouard, Frederic Bounoure, Nicolas Milon, Youness Karrout, Mohamed Skiba

**Affiliations:** 1 UFR of Health, Laboratory of Pharmaceutical & Biopharmaceutical technology, UNIROUEN, Normandy University, 76183 Rouen Cedex, France; francois.hallouard@gmail.com (F.H.); frederic.bounoure@univ-rouen.fr (F.B.); nicolas.milon@univ-rouen.fr (N.M.); 2 Univ. Lille, Inserm, CHU Lille, U1008, F-59000 Lille, France; youness.karrout@univ-lille.fr

**Keywords:** cyclosporine A, Poly-αβ-Cyclodextrin, spherical amorphous solid dispersion, enhanced bioavailability

## Abstract

Cyclosporine (CsA) has a selective property of suppressing various T-lymphocyte functions. This is of utmost importance in preventing allograft rejection by several organ transplantations, as well as in the treatment of systemic and local autoimmune disorders. However, the poor water solubility of CsA can be a major hurdle for its absorption into the blood stream, which leads to low bioavailability and thus less efficacy. The aim of this study was to prepare, characterize, and evaluate in vitro as well as in vivo, the potential of the innovative CsA drug delivery system. The latter contains CsA in spherical amorphous solid dispersion (SASD) which is embedded in an original α-cyclodextrin and β-cyclodextrin polymer mixture (Poly-αβ-CD) as a multifunctional amorphous carrier. The new developed SASD formulation showed that CsA was molecularly dispersed in αβ-cyclodextrins in an amorphous form, as was confirmed by physicochemical characterization studies. Interestingly, the peptide secondary structure, and thus, the drug activity was not impacted by the preparation of SASD as was shown by circular dichroism. Furthermore, the in vitro CsA release profile kinetics was almost identical to the commercially available product Neoral^®^. This study presents the first in vivo proof-of-concept for a novel drug delivery system based on Poly-αβ-CD containing CsA, with SASD allowing for increased bioavailibility. The pharmacokinetic parameters of cyclosporine A from the spherical spray-dried dispersion formulation was demonstrated in a “rat” animal model. For comparison, the commercially available Neoral® was studied. Importantly, the pharmacokinetic parameters were improved by extending *T*_max_ from 2 to 3 h after the oral administration in rats, and eventually preventing the enterohepatic circulation. All these results clearly demonstrate the improved pharmacokinetic parameters and enhanced bioavailability of CsA in the new developed drug delivery system. These data demonstrated the superiority of the newly developed Poly-αβ-CD formulation for oral administration of the poorly soluble CsA in vivo without altering its secondary structure. Poly-αβ-CD can be a very useful tool for the oral administration of poorly water-soluble drugs.

## 1. Introduction

Cyclosporine (CsA) is an oligopeptide with a molecular weight of 1202.64 Da, which in 1971 was for the first time isolated from crude extracts of the fungus *Tolypocladium inflatum gams* by Sandoz. This substance has the selective property of suppressing various T-lymphocyte functions, in particularly the production of interleukin-2 [2]. CsA is therefore used in hospitals for treatment since 1983, in preventing allograft rejection in various organ transplantations, and for the treatment of systemic and local autoimmune disorders [2,3]. 

CsA has significantly enhanced the initial, and also the long-term survival of transplant patients. However, the poor water solubility of this drug is a serious problem, and can consequently decrease the efficacy of the treatment due to low bioavailability, which strongly depends on drug-solubility in the gastrointestinal tract. [4]. Sandimmune^®^ and Neoral^®^ are the two commercially available products for the oral administration of this drug. They have been formulated in a self-emulsifying drug delivery systems “microemulsion” using soft or hard capsules. The main difference between these two formulations is the particle size distribution. Sandimmune^®^ droplet size ranges from few nanometers to several micrometers, while Neoral^®^ has a narrower monodisperse system with a size range between 100 and 250 nm [5]. This difference explains the great CsA bioavailability of Sandimmune^®^ ranging from 10 to 60% [1]. However, the main inconveniece of Neoral^®^ is its high content of surfactant, which is nonionic (polyoxyethylated castor oil, Kolliphor ELP^®^). This surfactant is well known to exert several serious side effects; such as hypersensivity, nephrotoxicity, and anaphylactoid reactions [6]. In addition, manufacturing soft or hard capsules is not harmless in the pharmaceutical industry.

Many approaches have been described in the literature in order to overcome the big problem of surfactants, which are to date mandatory for the CsA formulation (e.g., micronization, encapsulation in nanometric carriers [7], and solid dispersion methods [8]). Amorphous solid dispersions have many important advantages compared to other solubilization techniques. These advantages are robustness, reliability, and reproducibility that can be applied broadly to poor water-soluble compounds. Importantly, this solubilization method improves drug bioavailability due to the better wettability, increased porosity, and changing of polymorphic forms [9]. Furthermore, in solid dispersions the drug-carrier interactions prevent the agglomeration of drug particles, which hinders the free solubility of active pharmaceutical ingredients. It has to be pointed out that by using the solid dispersion technique, drug particle sizes can be easily reduced into molecular levels. However, other conventional techniques are able to reduce the particle size up to 2–5 µm. In addition, third-generation solid dispersions, including surfactants or complexing agents are also able to enhance the prevention of drug agglomeration. Complexing agents such as cyclodextrins or calixaren are particularly very interesting tools for maintaining poor water soluble drugs molecularly into such drug delivery systems by complexing them into the cavities of poly-cyclodextrins [9]. It has been shown that manufacturing solid dispersions can improve the solubility of drugs such as nimesulide, albendazole, and piroxicam [10,11,12]. 

In solid dispersions, cyclosporine is dispersed with polymers such as hydroxypropyl cellulose–SSL [13], dimethyl-β-cyclodextrin [13], hydroxypropyl methylcellulose phthalate and polyoxyethylene hydrogenated castor oil [14], polyoxyethylene (40) stearate [15], dimyristoyl phosphatidylcholine [16], and sodium lauryl sulfate and dextrin [17] in order to reach a better intrinsic solubility, dissolution rate, absorption rate, and thus, its oral bioavailability in vivo. 

Previously, we have invented and patented an original solvent-free process of producing cyclodextrin or calixarene based polymers [18]. Such high hydrophilic polymers which are freely water-soluble (>1g/ml) could have very interesting properties as multifunctional amorphous carrier for solid dispersions. Clearly, the presence of cyclodextrins or calixarenes as polymer constituents, allowing for drug inclusion in polymeric carriers, leads potentially to molecular and homogenous dispersion of drugs into the preparation. This can be attributed to the fact that drugs show high wettability and could not be re-crystallized in solid dispersion. 

The aim of this work was to develop and evaluate, in vitro as well as in vivo, spherical amorphous solid dispersion (SASD) using αβ-cyclodextrins polymers (Poly-αβ-CD). The main objectives were to: (i) Prepare a new developed CsA formulation with less excipients such as surfactants; (ii) improve the bioavailability as well as the efficacy of the poorly water-soluble cyclosporine; and (iii) characterize and identify physico-chemically the stable form of CsA into the solid amorphous dispersion (SASD/CsA), avoiding crystallization or phase separation of the amorphous form. Moreover, the physicochemical properties of CsA, Poly-αβ-CD, physical mixture of Poly-αβ-CD/CsA and SASD/CsA were characterized in terms of morphology, particle size distribution, crystallinity, dissolution behavior, and the peptide secondary structure was evaluated by circular dichroism. Oral absorption of CsA was demonstrated and evaluated after oral administration of SASD/CsA in rats. For comparative analysis, the commercially available product Neoral^®^ was also evaluated in vivo. 

## 2. Materials and Methods

### 2.1. Chemicals

α-cyclodextrins, and β-cyclodextrins (Wacker, Munich, Germany). Sodium chloride, citric acid, pepsin, sodium phosphate dibasic, and crystalline cyclosporine extra pure (Sigma Aldrich, Saint-Louis, MO, USA). *N*,*N*-dimethyldodecylamine-*N*-oxide-30% (LDAO) in water (Molekula, Gillingham, United Kingdom). Ethanol, methanol, heptanes, dichloromethane, and acetonitrile (VWR, Radnor, PA, USA). The polymer of cyclodextrins (Poly-αβ-CD) was synthesized according to the process invented and patented by Skiba [18]. 

### 2.2. Synthesis of Terpolymers of CD (Poly-αβ-CD)

Cyclodextrin polymers were synthesized using a direct melt polycondensation process according to the method reported by Skiba [17]. Briefly, a mixture of known amount (*w*/*w*) of cyclodextrins (α and β-CD), citric acid, and sodium phosphate dibasic was transferred into a reactor, which was maintained at temperature ranging between 140 °C and 150 °C for a fixed duration of time. The obtained solid form was dissolved in water and dialyzed using a polyether sulfate membrane filter with molecular weight cut off of 10,000 Da. After the dialysis, the resulting solution was spray-dried with a Mini Spray Dryer B-290^®^ (BÜCHI, Flawil, Switzerland). 

### 2.3. Preparation of Cyclosporin A—Spherical Amorphous Solid Dispersion 

Cyclosporine-loaded in SASD was prepared by the spray-drying technique using a Buchi 290 nozzle type mini spray dryer (Flawil, Switzerland). The spray-dried dispersions were prepared as follows: 0.3–1 g of cyclosporine was added to an aqueous solution of 3–10 g of poly-cyclodextrins. The aforementioned polymer was separately dissolved in 300 mL of purified water then cyclosporine was subsequently mixed with the solution until a homogeneous dispersion was obtained. The process parameters of spray-drying were as follows: Inlet temperature = 150 °C; outlet temperature = 80–90 °C; flow rate of the pump = 20%; nozzle diameter = 1.4 mm. The flow rate of the drying air was maintained at the aspirator setting of 50, which indicated the pressure of the aspirator filter vessel and this was set at −40 mbar. The direction of air flow was the same as that of sprayed products. 

### 2.4. Measurements of Particle Size and Polydispersity

The hydrodynamic diameter and Polydispersity index (PDI) were obtained by dynamic light scattering using a DLS instrument (Mastersizer 2000S^®^, Malvern, Orsay, France). SPAN factor was defined as follows: SPAN = (*d*_90_-*d*_10_)/*d*_50_ where *d*_10_, *d*_50_ and *d*_90_ are the particle diameters at 10%, 50%, and 90% of cumulative volume, respectively. SPAN was calculated in order to illustrate particle size uniformity. The mean particle size was measured after performing the experiment for each batch three times.

### 2.5. Appearance of Surface Morphology

Surface morphology of SASD in the powder form were visualized using a JCM-5000 NeoScope^®^ microscope (JEOL, Akishima-Shi, Tokyo, Japan), at an accelerated voltage between 10 and 15 kV. Powder samples were stuck on a SEM stub with 146 conductive adhesive tapes and coated with gold to reduce electric charges, which is induced during analysis with a NeoCoater MP-19020NCTR^®^ (JEOL, Akishima-Shi, Tokyo, Japan).

### 2.6. Measurement of Crystallinity

#### 2.6.1. Powder X-ray Diffraction

The physical state “crystal/amorphous” of the drug as well as polymers was studied by an X-ray powder diffractometer (XRPD) D8 Discover^®^ (Bruker, Billerica, MA, USA) equipped with the manufacturer software version 2.6.1. The instrument was equipped with a X-ray tube, containing a copper anticathode (40 kV, 40 mA, Kα1 radiation: 1.5406Å, Kα2 radiation: 1.5444Å) and mounted with an angular detector—Lynx eye™ (Bruker, Billerica, MA, USA). The scan step was fixed at ~0.04° with a counting time of 0.5 sec/step over an angular range 3°–30°.

#### 2.6.2. Nuclear Magnetic Resonance

The solid-state ^13^C NMR experiments were performed on an AV-400^®^ spectrometer (Bruker, Billerica, MA, USA), which was equipped with a probe of 4 mm MAS BB with rotation at 12500 Hz (MAS), CP3lev with ramp up between 60 to 100% (contact time: Tcp of 3.5 ms, contact strength ^13^C of 45 Hz, contact strength 1H with polarization rump between 35 to 60 kHz) and decoupled proton type spinal 64 (~60 kHz), and samples were packed into 5.0 mm o.d. glass tubes.

### 2.7. Circular Dichroism Spectroscopy 

Circular dichroism (CD) spectra recorded at room temperature from 190 to 260 nm on a CD6 dichrograph (Jobin-Yvon, Longjumeau, FRA), using quartz cells with a 0.05 cm path length. For each spectrum, 5 scans were accumulated and then averaged. The baseline was obtained by recording a spectrum of the neat solution, and it was subtracted from the peptide spectrum. Ellipticity was reported as mean residue molar ellipticity in deg·cm^2^·dmol^−1^. Crystalline CsA and also microspheres formulations equivalent to 0.4 mg/mL of CsA was dissolved in diluted acetonitrile with water (55% *w*/*w*), and analyzed for secondary structure where the CD spectra were accumulated three times for data collection. Each data point was carried out in triplicate.

### 2.8. In Vitro Dissolution test

In vitro CsA dissolution was performed using a USP dissolution apparatus (paddle, VK7000^®^, Varian/Vankel, Palo Alto, CA, USA). Soft gelatin capsules (25 mg) of the commercially available product Neoral^®^ (Novartis, Bâle, Switzerland), or Sandimmune^®^ (Novartis, Bâle, Switzerland) were put into a sinker prior introducing to the release medium. CsA-loaded spray-dried dispersion formulations equivalent to 25 mg of CsA were directly introduced to the release medium (500 mL, 0.4% *v*/*v* LDAO in water, simulated gastric fluid with pepsin at pH 1.2, 37 ± 0.5 °C, paddle speed at 100 rpm. One mL sample was withdrawn without medium replacement at 10, 20, 30, 45, 60, and 90 min and subsequently measured by HPLC at a wavelength of 212 nm for cyclosporine A content. 

### 2.9. Measurements of Pharmacokinetic Parameters and Bioavailability 

An in vivo study with male Sprague–Dawley rats was conducted in animal facilities respecting all governmental guidelines and ethical rules, and in accordance with the European Union regulations and the agreement of the Committee on Animal Research and Ethics of Rouen University. 

For this study, 6 male Sprague–Dawley (SD) rats weighing 250 ± 10 g were selected. Three animals were housed per cage before the experimental study, respecting 12:12 h dark/light cycle, all rats had free access to standard chow and tap water. Prior to the oral administration, animals were fasted overnight and had access to water ad libitum. A single CsA oral dose (10 mg/kg [20]) of either Neoral^®^ or SASD/CsA was given to each animal by gavages between 09:00 and 10:00 a.m. 

#### 2.9.1. Cyclosporine Quantification in Blood Plasma of Rats 

Blood samples (0.3 mL) were withdrawn from rat tails and analyzed for their drug contents. Rats were anesthetized by inhalation using Halothane. Blood samples were collected in heparin vials after 1, 2, 3, 4, 5, and 6 h of the oral administration. These samples were subsequently mixed and centrifuged at 10,000 rpm for 5 min at 5 °C. Samples of plasma were frozen at −20 °C until analysis. 

The CsA contents of plasma samples analyzed by the HPLC system model 1050 (Hewlett-Packard, Palo Alto, CA, USA). Mobile phase was acetonitrile:water (85:15). The separation was achieved using a reversed-phase column [NUCLEOSIL 120-10^®^ (C_18_, 10 mm) from Macherey-Nagel & Co (Düren, Germany)]. The flow rate was 1.0 ml/min, and the column was maintained at 70 °C. The drug content was measured at a wavelength of 210 nm.

A solid phase extraction was used to extract cyclosporine from blood plasma. The conditioning of the C_18_ (MEPS) cartridge was done with dichloromethane, followed by pure acetonitrile, 50% acetonitrile:water, and 80% acetonitrile:water. A 250 μL syringe, fitted with a C_18_ cartridge, was used for the HPLC analysis, removing 100 μl of plasma samples, and then the aqueous portion was discarded through the C_18_, which led to the cyclosporine adsorption into the solid phase. The C_18_ was washed 3 times with 250 μL of water, and subsequently, cyclosporine (50 μL) was eluted by pure acetonitrile. The sensitivity of this method was 20 ng/mL and the linearity ranged from 30 ng/mL to 10 μg/mL.

#### 2.9.2. Statistical Analysis 

Pharmacokinetic analysis of plasma concentration results was performed using model independent methods. The area under the curve of the plasma concentration-time (AUC) and the cumulative AUC were assessed by the WinNonlin program (version 5.2 Pharsight Corp., Mountain View, CA), using the non-compartment method. 

Maximum whole blood concentration (*C*_max_) and time to reach maximum whole blood concentration (*T*_max_) were directly obtained from the plotted data. The relative oral bioavailability for the SASD/CsA formulation was determined by the ratio of AUC**_SASD/_**AUC**_Neoral®_**, and was expressed as a percentage.

## 3. Results and Discussion

The aim of this work was to achieve an easy, quick, and reproducible manufacturing process for the oral administration of poor water-soluble drugs such as cyclosporine A. These formulations should preferably have high drug in order to reduce the volume of oral preparation. On one hand, the compliance will be increased, on the other hand this will help the elderly overcome deglutination problem. In additions, drugs should present high bioavailability and potentially suppress the enterohepatic circulation of CsA. The drug concentration adjustment can be facilitated for personalized medicines. 

To fulfill these objectives, we used amorphous solid dispersion in order to achieve orally high drug bioavailability due to the high soluble amorphous carriers, which led to the release of the drug with surpersaturation in the dissolution medium. This allowed to simultaneously increase drug solubility, as well as decrease the formulation shape. In addition, this work illustrated how to perform and maintain molecular drugs into solid dispersion, in a stable form, to avoide drug crystallization. This was achieved by optimizing drug solubility and wettability using the drug-cyclodextrin inclusion form by means of our invented and patented Poly-αβ- cyclodextrins. 

### 3.1. Characterization of Spray-Dried Dispersions 

#### 3.1.1. Particle Size and Distribution of SASD/CsA 

The particle size distribution of cyclosporine-loaded SASD was analyzed by Malvern 2000S. A narrow distribution centered on two particle populations was observed. The main population should be a mean particle size of 18 µm (Table 1).

Particle size of cyclosporine-loaded SASD had a few influences on the feasibility of per os administration. Clearly, pharmaceutical solid dosage forms can be administered up to several “cm” such as tablets via oral route. However, the particle size contrary to the shape of the dosage form has a great influence on drug solubility and thus drug bioavailability. The diffusion coefficient is inversely proportional to the radius of microspheres as shown in the Stokes–Einstein equation. That is why the diffusion coefficient was heavily increased and as microspheres size was reduced, the dissolution rate of the drug became faster. This was described by the particle size distribution data of SASD/CsA formulation.

(1) $$D=\frac{R\cdot T}{6\pi \cdot \eta \cdot r\cdot N}$$

The equation’s terms are defined as follows: *R*: Molar gas constant; *T*: Absolute temperature; *η*: Apparent viscosity; *r*: Radius of a microsphere; *N*: Avogadro’s number.

#### 3.1.2. Surface Morphology 

**Figure 1:**
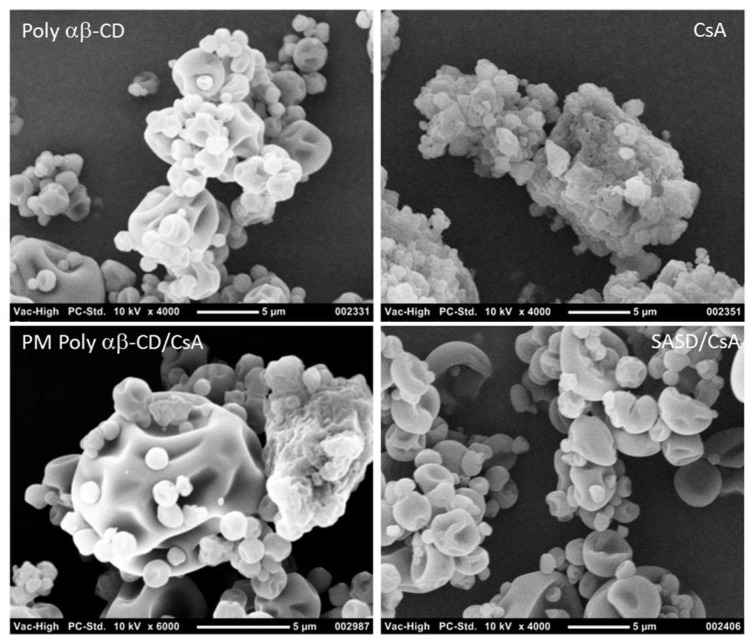
SEM micrographs from Poly-αβ-CD copolymer (top left), crystalline cyclosporine (CsA; top right), physical mixture of Poly-αβ-CD/CsA (bottom left), and spherical amorphous solid dispersion based on Poly-αβ-CD copolymer of CsA (SASD/CsA; bottom right).

To confirm and better understand these results, SEM observations of Poly-αβ-CD, crystalline CsA, physical mixture of Poly-αβ-CD:CsA, and SASD of CsA were performed (Figure 1). 

**Figure 2:**
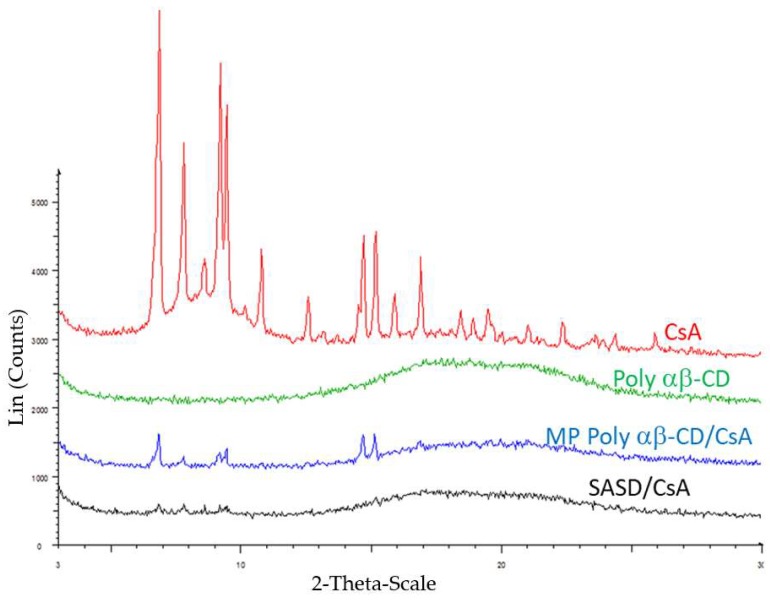
X-ray diffraction patterns of crystalline cyclosporine (CsA), Poly-αβ-CD copolymer, physical mixture of Poly-αβ-CD/CsA, and spherical amorphous solid dispersion based on Poly-αβ-CD copolymer of CsA (SASD/CsA). The curves are illustrated respectively from top to bottom as indicated in the diagram.

As it can be seen in Figure 2, SEM pictures of SASD/CsA confirmed the presence of a principal population of particles having a mean size over 10 µm, and a minor population of particles having approximately a mean size of 1 µm. SEM observations revealed also a clear morphological change between SASD/CsA and crystalline CsA or the physical mixture of Poly-αβ-CD:CsA. Interestingly, in SASD/CsA formulations, the dissolved polymer was adsorbed to the surface of the dispersed cyclosporine A and hindered its re-crystallization. This can be attributed to the fact that the presence of the polymer “Poly-αβ-CD” was enough to solubilize the poor water-soluble drug into the matrices and also play a major role in the induction of the amorphous form of cyclosporine A. In addition, the solid dispersion process of drug in Poly-αβ-CD was very useful for producing a stable amorphous form of poor water-soluble drugs.

### 3.2. Crystallinity

#### 3.2.1. Powder X-ray Diffraction

The amorphous form of a hydrophobic drug has a significantly higher free energy than the crystalline form, and this why it is expected to have a much higher aqueous solubility [21,22]. However, this drug form being thermodynamically metastable has a tendency to precipitate or re-crystallize. To prevent/delay this phenomenon, drug-polycyclodextrin inclusions have been described in the literature showing interesting outcomes concerning the improvement of drug solubility. 

PXRD and ^13^C CPMAS NMR spectral analysis were then performed to investigate the drug form in Pol-αβ-CD solid dispersion formulation. Figure 2 illustrates the PXRD spectra of Poly-αβ-CD, crystalline CsA, physical mixture of Poly-αβ-CD:CsA, and SASD/CsA. Several and analogous intense peaks were observed for CsA and also the physical mixture of Poly-α β-CD:CsA which indicated that CsA in the pure as well as in the physical mixture was in the crystalline form. However, in the SASD formulation it exhibited a halo diffraction pattern, and also the absence of such peaks by Poly-αβ-CD polymer. These results indicated that CsA in SASD formulation as well as the pure Poly-αβ-CD copolymer was in the amorphous form. In addition, PXRD spectrum of the crystalline CsA was clearly indicative of a tetragonal crystal form [23].

#### 3.2.2. Nuclear Magnetic Resonance 

**Figure 3:**
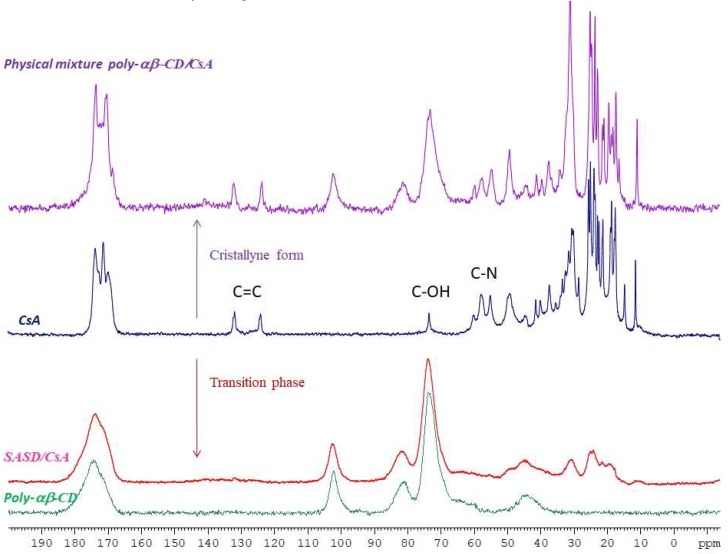
^13^C CPMAS NMR spectra of crystalline cyclosporine A (CsA; blue color), Poly-αβ-CD (green color), physical mixture of Poly-αβ-CD/CsA (purple color), and spherical amorphous solid dispersion based on Poly-αβ-CD copolymer of CsA (SASD/CsA; red color) as indicated in the diagram.

^13^C CPMAS NMR spectral analysis of Poly-αβ-CD (Figure 3), crystalline CsA, physical mixture of Poly-α-CD:CsA, and SASD/CsA are illustrated in Figure 3. The physical mixture Poly-αβ-CD:CsA showed the same spectra as cyclosporine alone with intense alkyl C–C peaks of chemical shift between 30 and 10, N–C=O at 174–170; C=O at 130–120; C–OH at 75-70 and C–N at 60–50 ppm, which indicated that cyclosporine preserved its crystalline form in the physical mixture with the Poly-αβ-CD. However, in spray-dried dispersion formulation, the intensity of alkyl C-C along with the chemical shifts was dramatically changed.

This phenomenon was confirmed by the SEM and PXRD analysis (Figure 1 and Figure 2). 

An interaction of the drug with Poly-αβ-CD polymer in SASD may be the cause of cyclosporine amorphization. This hypothesis is in concordance with ^13^C CPMAS NMR spectral analysis. The intensity of alkyl C–C along with the chemical shifts was changed for the spray-dried dispersion formulation. This last indicated a hydrophobic interaction between cyclosporine and copolymer. 

These drug-polymer interactions render the cyclosporine molecules more soluble, which can increase significantly the bioavailability in vivo.

Importantly, circular dichroism analysis revealed that the peptide nature of CsA remains stable even after the manufacturing process of the solid dispersion by spray drying. It has been shown that the secondary structure of CsA in Poly-αβ-CD SASD formulation was not impacted, which is of utmost importance in the cyclosporine A treatment efficacy. 

### 3.3. Circular Dichroism Spectroscopy Property

**Figure 4:**
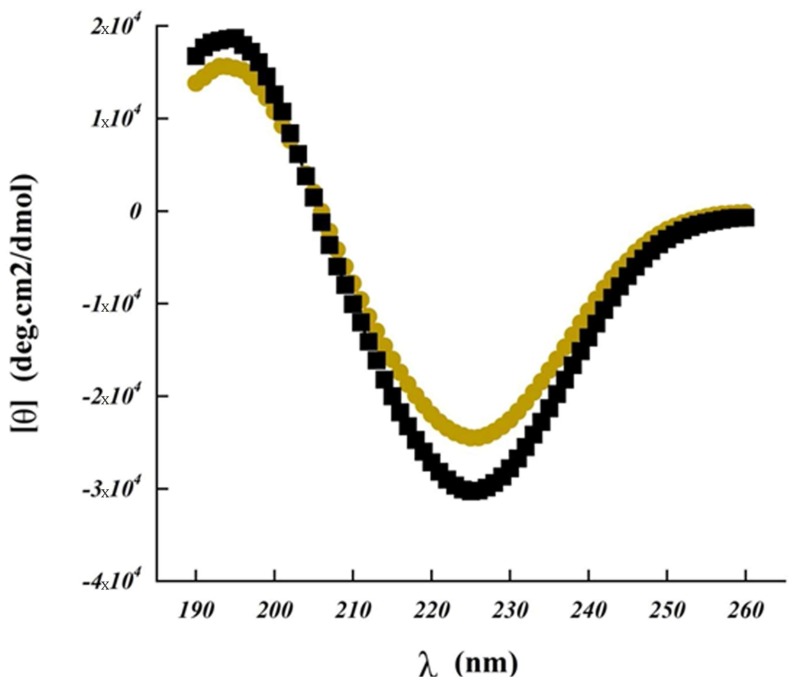
Far ultra-violet circular dichroism spectrum of cyclosporine (CsA) upon aqueous diluted solution with 55% of acetonitrile, and spherical amorphous solid dispersion based on Poly-αβ-CD polymer of CsA (SASD/CsA). Samples were measured in triplicate.

CD spectroscopy (Figure 4) measures differences in the absorption of left-handed polarized versus right-handed polarized light, which arises due to the structural asymmetry.

The absence of regular structure results in zero CD intensity, while an ordered structure results in a spectrum which contains positive and negative signals [24,25]. Bands measured at 190–260 nm corresponding to the peptide bonds and potential side chains of other molecules present during this analysis (Figure 4). These results showed us the secondary structure. Bands measured at 206–300 nm correspond to amino acids and cysteine which can be utilized to determine the tertiary structure [26]. Like all spectroscopic techniques, the CD signal reflects an average of the entire molecular population. For CsA and for SASD/CsA, we observed a long-wavelength minimum occuring near 225 nm, with an ellipticity of approximately −25,000, accompanied by a maximum near 194 nm having an ellipticity of 16,000. These results were due to cyclosporine as β-turns, which is in accordance with other cyclic peptides such as the CD spectrum for Cyclo (l-Om-l-Pro-d-Phe) [27]. Concerning the influence of different ratios of copolymers:CsA (e.g. 3:1, 6:1, and 10:1), the obtained CD spectra were found to coincide with pure CsA. This showed that the secondary structure of cyclosporine was not, or only slightly, modified in the presence of the studied polymers (data not shown). Importantly, the drug entrapment in Poly-αβ-CD SASD formulation led to only minor differences in the cyclosporine circular dichroism spectrum, indicating that the CsA secondary structure was not impacted and remained stable without substantial alteration. Thus, this formulation seemed to be the appropriate carrier for cyclosporine, protecting its activity. 

### 3.4. In Vitro Dissolution Test 

**Figure 5:**
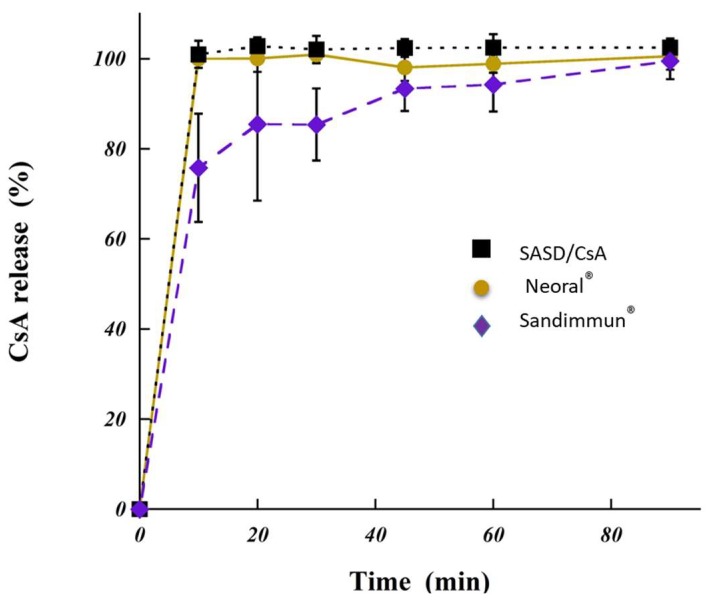
In vitro dissolution of cyclosporine A (CsA) from spherical amorphous solid dispersion based on Poly-αβ-CD polymer (SASD/CsA) under conditions simulating the gastric fluid. The point at zero minute is theoretical. For reasons of comparison, two different commercially available products under conditions simulating the gastric fluid are shown; Neoral^®^ and Sandimmun^®^^.^

When developing oral amorphous solid dispersion for hydrophobic drugs such as CsA (SASD/CsA), it is important to control the dissolution rate in order to avoid precipitation upon dilution in the gastrointestinal tract and maximize the absorption in the intestine. The relative dissolution rate of CsA from SASD formulation, Neoral^®^ and Sandimmune^®^ are shown in Figure 5. Immediate cyclosporine A dissolution (100%) from SASD/CsA formulation and Neoral^®^ was observed after 10 min, while only 76% of CsA from Sandimmune^®^. In addition, the CsA dissolution profile of Sandimmune^®^ presented after 30 min a progressive increase in the drug dissolution rate reaching 95% CsA after 90 min. 

The immediate drug dissolution from SASD formulation confirmed, as expected, the physicochemical characterization of the newly developed formulation. The increase in the dissolution rate could be explained by the absence of drug crystallinity and presence of the highly water soluble amorphous form [28], as illustrated previously in this work by our physicochemical studies. Amazingly, the high aqueous solubility of Poly-αβ-CD (greater than 1g/ml) led to better wetting of drug particles and thus increased solubility [18]. It is to emphasize that the new developed formulation based on SASD-containing CsA showed significantly better results and higher drug release rate compared to solid dispersion based on hydroxypropyl cellulose HPC (SSL)- or polyethylene glycol (PEG-6000)-containing CsA [29,30]. This data was also consistent with previous works reporting an increase in the dissolution rate of hydrophobic drug, which was molecularly dispersed as in the case of solid dispersion [31,32,33]. 

### 3.5. Bioavailability and Pharmacokinetic Parameters

To evaluate the pharmacokinetics parameters of the proposed SASD/CsA formulation to improve the oral bioavailability of cyclosporine A, the SASD/CsA formulation was orally administered to Sprague–Dawley rats. Importantly, animals did not express any pain or discomfort during the experiment. In addition, no weight loss was detected, which indicated good tolerance of this potential medicine. It has to be pointed out that the drug as well as the polymer “Poly-αβ-CD” can be used in drug delivery systems without any risk of toxicity.

**Figure 6:**
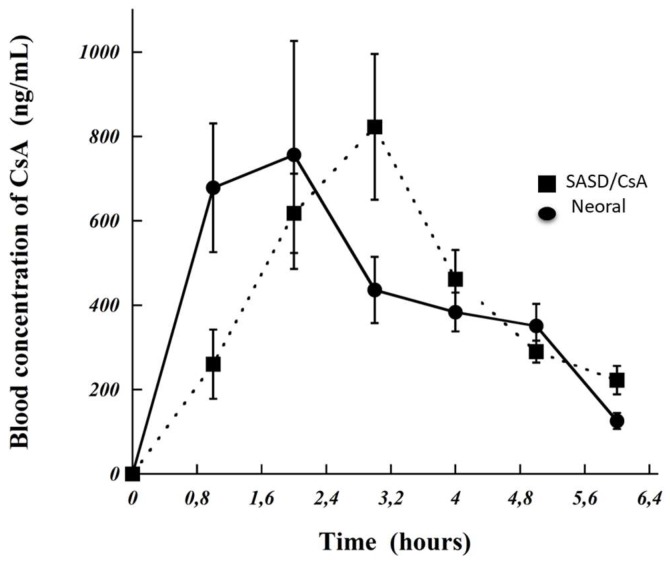
Cyclosporine A (CsA) concentrations in the plasma of rats treated orally with Neoral^®^ and spherical amorphous solid dispersion based on Poly-αβ-CD copolymers (SASD/CsA). Rats received CsA (10 mg/kg) and for each product. Three rats were used.

As it can be seen in Figure 6, the *C*_max_ and *T*_max_ values were increased by 9 and 50%, respectively, while AUC was almost identical to Neoral (Table 2 and Figure 6). This indicated that the newly developed formulation “SASD/CsA based on Poly-αβ-CD” shows very promising results in vivo. These data were also higher than those observed with the native α-cyclodextrin [34]. Unlike nanometric formulations, the pharmacokinetic parameter “*T*_max_“ increased [35], which can probably attributed to the slow solubility of cyclosporine particles (e.g., poor moistening ability). However, the absorption rate of cyclosporine from Neoral^®^ was superior to the one of SASD/CsA. In clinic, *C*_max_ and *T*_max_ modifications compared to Neoral^®^ will not be deleterious. Clearly, the toxicity risk induced by the increase of *C*_max_ can be easily prevented by reducing the concentration of CsA in the SASD/CsA formulation. Concerning *T*_max_ extension, a short delay effect of this drug was not required in front of CsA indication and administration route. For orally administered maintenance treatment, an extension of *T*_max_ was conversely favorable, which allowed for a stable steady-state of the drug in blood.

An ideal drug delivery for CsA for optimized systemic drug concentration in order to maximize the therapeutic efficacy should not only improve the bioavailability and minimize serious side effects, but should also avoid the enterohepatic circulation of CsA. This phenomenon was observed with the newly developed formulation “SASD/CsA”, contrary to the nanometric formulation [35]. This can be probably attributed to the fact that the enterohepatic circulation with the newly developed formulation was shifted due to the reduced drug concentration into the formulation and also the decreased quantity of the drug administered orally to rats. A second hypothesis is that SASD/CsA is highly hydrophilic and can be freely solubilized in blood which can be consequently eliminated by the kidneys in the case of the new developed formulation “SASD/CsA” shunting hepatic elimination predominant for Neoral^®^ formulation (about 90%) [36]. This very interesting phenomenon can prevent the enterohepatic circulation, which can be very beneficial for patients reducing serious side effects caused by cyclosporine A. 

In the hospital, avoiding the first pass effect can be seen as a very interesting phenomenon for some drugs. Cyclosporine A is well known for the interactions with a multitude of drugs or aliments, which induce CYP3A4 and affect P-glycoprotein. Cyclosporine dosage forms should, therefore, be individually prepared and adjusted for their drug content in particular for pediatric onco-haematology in order to prevent any severe graft-versus-host disease caused by the drug toxicity in the case of overdose or the failure of the treatment due to the under dosing [37]. Bypass enterohepatic circulation can better control variability of CsA concentration in the blood stream. It is to emphasize that the newly developed formulation of CsA based on Poly-αβ-cyclodextrins “Spherical Amorphous Solid Dispersion” can be a very useful tool in the prediction of CsA blood-plasma concentration. In addition, this novel drug delivery system can be very helpful to optimize the appropriate drug concentration to be administered to children, adults, as well as the elderly. 

## 4. Conclusions

Poly-αβ-CD copolymers as matrices in spray-dried dispersion formulation containing cyclosporine A were developed and characterized in vitro as well as in vivo. The newly developed SASD formulation showed great enhancement of CsA solubility in aqueous medium by a factor of 9.8 due to the creation of an amorphous drug form and without altering the peptide secondary structure of the peptide “activity remains stable”. In addition, the particle size and thus the drug wettability were improved. Interestingly, in vitro release profile was almost similar to Neoral^®^ and faster than Sandimmune^®^. In vivo studies proved the improvement of the pharmacokinetic parameters after the oral administration, and also the absence of the acute toxicity preventing enterohepatic circulation. These results clearly prove the superiority of the proposed drug delivery system based on multifunctional amorphous polymer as a solid dispersion carrier for the development of dosage forms containing poor water-soluble drugs. 

## Figures and Tables

**Table 1:** Particle size distribution of cyclosporine A (CsA) in spherical amorphous solid dispersion formulation based on Poly-αβ-CD copolymers. Each measurement was carried out in triplicate.

Particle Diameter	SASD/CsA
D10 (μm)	1.6 ± 0.08
D50 (μm)	10.4 ± 0.22
D90 (μm)	18.8 ± 0.15

**Table 2:** Pharmacokinetic parameters of cyclosporine A (CsA) after the oral administration of Neoral® (cyclosporine A in a microemulsion) or spherical amorphous solid dispersion based on Poly-αβ-CD copolymer containing CsA (SASD/CsA).

Parameters	Neoral^®^	SASD/CsA	AUC_SASD/_AUC_Neoral®_
*C*_max_ (ng/mL)	756.35	822.89	-
AUC_0–6hr_ (ng·h/mL)	2669	2564	96%
AUC_0–∞_ (ng·h/mL)	2791	3402	122%
*T*_max_ (h)	2	3	-

## References

[1] Stähelin H.F. (1996). The history of cyclosporin A (Sandimmune) revisited: Another point of view. Experientia.

[2] Dunn C.J., Wagstaff A.J., Perry C.M., Plosker G.L., Goa K.L. (2001). Cyclosporin: An updated review of the pharmacokinetic properties, clinical efficacy and tolerability of a microemulsion-based formulation (neoral)1 in organ transplantation. Drugs.

[3] Zijlstra G.S., Rijkeboer M., Jan van Drooge D., Sutter M., Jiskoot W., van de Weert M., Hinrichs W.L.J., Frijlink H.W. (2007). Characterization of a cyclosporine solid dispersion for inhalation. AAPS J..

[4] Chiu Y.-Y., Higaki K., Neudeck B.L., Barnett J.L., Welage L.S., Amidon G.L. (2003). Human Jejunal Permeability of Cyclosporin A: Influence of Surfactants on P-Glycoprotein Efflux in Caco-2 Cells. Pharm. Res..

[5] Neslihan Gursoy R., Benita S. (2004). Self-emulsifying drug delivery systems (SEDDS) for improved oral delivery of lipophilic drugs. Biomed. Pharm..

[6] Luke D.R., Kasiske B.L., Matzke G.R., Awni W.M., Keane W.F. (1987). Effects of cyclosporine on the isolated perfused rat kidney. Transplantation.

[7] Guada M., Lasa-Saracíbar B., Lana H., del Carmen Dios-Viéitez M., Blanco-Prieto M.J. (2016). Lipid nanoparticles enhance the absorption of cyclosporine A through the gastrointestinal barrier: In vitro and in vivo studies. Int. J. Pharm..

[8] Nazzal S., Guven N., Reddy I.K., Khan M.A. (2002). Preparation and Characterization of Coenzyme Q10–Eudragit^®^ Solid Dispersion. Drug Dev. Ind. Pharm..

[9] Vo C.L.-N., Park C., Lee B.-J. (2013). Current trends and future perspectives of solid dispersions containing poorly water-soluble drugs. Eur. J. Pharm. Biopharm..

[10] Joudieh S., Lahiani-Skiba M., Bon P., Ba O., Le Breton J., Skiba M. (2008). Nimesulide Apparent Solubility Enhancement with Natural Cyclodextrins and their Polymers. Lett. Drug Des. Discov..

[11] Joudieh S., Bon P., Martel B., Skiba M., Lahiani-Skiba M. (2009). Cyclodextrin polymers as efficient solubilizers of albendazole: Complexation and physico-chemical characterization. J. Nanosci. Nanotechnol..

[12] Bouchal F., Skiba M., Chaffai N., Hallouard F., Fatmi S., Lahiani-Skiba M. (2015). Fast dissolving cyclodextrin complex of piroxicam in solid dispersion Part I: Influence of β-CD and HPβ-CD on the dissolution rate of piroxicam. Int. J. Pharm..

[13] Chen H., Jiang G., Ding F. (2009). Monolithic Osmotic Tablet Containing Solid Dispersion of 10-hydroxycamptothecin. Drug Dev. Ind. Pharm..

[14] Kanagale P., Patel V., Venkatesan N., Jain M., Patel P., Misra A. (2008). Pharmaceutical Development of Solid Dispersion Based Osmotic Drug Delivery System for Nifedipine. Curr. Drug Deliv..

[15] Liu C., Zhu S.J., Zhou Y., Wei Y.P., Pei Y.Y. (2006). Enhancement of dissolution of cyclosporine A using solid dispersions with polyoxyethylene (40) stearate. Pharmazie.

[16] Zidan A.S., Habib M.J., Khan M.A. (2008). Process analytical technology: Nondestructive evaluation of cyclosporine A and phospholipid solid dispersions by near infrared spectroscopy and imaging. J. Pharm. Sci..

[17] Lee E.-J., Lee S.-W., Choi H.-G., Kim C.-K. (2001). Bioavailability of cyclosporin A dispersed in sodium lauryl sulfate–dextrin based solid microspheres. Int. J. Pharm..

[18] Skiba M. (2010). Method for Synthesizing Calixarene and/or Cyclodextrin Copolymers, Terpolymers and Tetrapolymers, and Uses Thereof.

[19] Skiba M., Lahiani-Skiba M. (2013). Novel method for preparation of cyclodextrin polymers: Physico-chemical characterization and cytotoxicity. J. Incl. Phenom. Macrocycl. Chem..

[20] da Silva Peralta F., Pallos D., Silva Queiroz C., Ricardo L.H. (2015). Previous exposure to Cyclosporine A and periodontal breakdown in rats. Arch. Oral Biol..

[21] Zanotto E.D. (1998). Do cathedral glasses flow?. Am. J. Phys..

[22] Serajuddin A.T. (1999). Solid dispersion of poorly water-soluble drugs: Early promises, subsequent problems, and recent breakthroughs. J. Pharm. Sci..

[23] Yamashita K., Nakate T., Okimoto K., Ohike A., Tokunaga Y., Ibuki R., Higaki K., Kimura T. (2003). Establishment of new preparation method for solid dispersion formulation of tacrolimus. Int. J. Pharm..

[24] Greenfield N.J. (1996). Methods to estimate the conformation of proteins and polypeptides from circular dichroism data. Anal. Biochem..

[25] Ng K., Zhao L., Meyer J.D., Rittmann-Grauer L., Manning M.C. (1997). Use of circular dichroism spectroscopy in determining the conformation of a monoclonal antibody prior to its incorporation in an immunoliposome. J. Pharm. Biomed. Anal..

[26] Righetti P.G., Verzola B. (2001). Folding/unfolding/refolding of proteins: Present methodologies in comparison with capillary zone electrophoresis. Electrophoresis.

[27] Brahms S., Brahms J., Spach G., Brack A. (1977). Identification of β,β-turns and unordered conformations in polypeptide chains by vacuum ultraviolet circular dichroism. Proc. Natl. Acad. Sci. USA.

[28] Leuner C., Dressman J. (2000). Improving drug solubility for oral delivery using solid dispersions. Eur. J. Pharm. Biopharm..

[29] Onoue S., Sato H., Ogawa K., Kawabata Y., Mizumoto T., Yuminoki K., Hashimoto N., Yamada S. (2010). Improved dissolution and pharmacokinetic behavior of cyclosporine A using high-energy amorphous solid dispersion approach. Int. J. Pharm..

[30] Rahman Z., Zidan A.S., Khan M.A. (2010). Formulation and evaluation of a protein-loaded solid dispersions by non-destructive methods. AAPS J..

[31] Dutet J., Lahiani-Skiba M., Didier L., Jezequel S., Bounoure F., Barbot C., Arnaud P., Skiba M. (2007). Nimesulide/cyclodextrin/PEG 6000 ternary complexes: Physico-chemical characterization, dissolution studies and bioavailability in rats. J. Incl. Phenom. Macrocycl. Chem..

[32] Lahiani-Skiba M., Barbot C., Bounoure F., Joudieh S., Skiba M. (2006). Solubility and dissolution rate of progesterone-cyclodextrin-polymer systems. Drug Dev. Ind. Pharm..

[33] Ren F., Jing Q., Tang Y., Shen Y., Chen J., Gao F., Cui J. (2006). Characteristics of bicalutamide solid dispersions and improvement of the dissolution. Drug Dev. Ind. Pharm..

[34] Miyake K., Irie T., Hirayama F., Uekama K., Labandeira J.J.T., Vila-Jato J.L. (1999). Improved Solubility and Oral Bioavailability of Cyclosporin a by Hydrophilic Cyclodextrin Complexation. Proceedings of the Ninth International Symposium on Cyclodextrins, Santiago de Compostela.

[35] Wang K., Qi J., Weng T., Tian Z., Lu Y., Hu K., Yin Z., Wu W. (2014). Enhancement of oral bioavailability of cyclosporine A: Comparison of various nanoscale drug-delivery systems. Int. J. Nanomed..

[36] (2014). Agence Nationale de Sécurité du Médicament et des Produits de Santé (ANSM) Product Characteristics Summary of Neoral. http://base-donnees-publique.medicaments.gouv.fr/affichageDoc.php?specid=65922428&typedoc=R.

[37] Bleyzac N. (2008). The use of pharmacokinetic models in paediatric onco-haematology: Effects on clinical outcome through the examples of busulfan and cyclosporine. Fundam. Clin. Pharmacol..

